# Noninvasive Prediction of Occult Peritoneal Metastasis in Gastric Cancer Using Deep Learning

**DOI:** 10.1001/jamanetworkopen.2020.32269

**Published:** 2021-01-05

**Authors:** Yuming Jiang, Xiaokun Liang, Wei Wang, Chuanli Chen, Qingyu Yuan, Xiaodong Zhang, Na Li, Hao Chen, Jiang Yu, Yaoqin Xie, Yikai Xu, Zhiwei Zhou, Guoxin Li, Ruijiang Li

**Affiliations:** 1 Department of Radiation Oncology, Stanford University School of Medicine, Stanford, California; 2 Shenzhen Institutes of Advanced Technology, Chinese Academy of Sciences, Shenzhen, Guangdong, China; 3 Shenzhen Colleges of Advanced Technology, University of Chinese Academy of Sciences, Shenzhen, Guangdong, China; 4 Department of Gastric Surgery, Sun Yat-sen University Cancer Center, Guangzhou, China; 5 Department of Medical Imaging Center, Nanfang Hospital, Southern Medical University, Guangzhou, China; 6 Department of Radiology, The Third Affiliated Hospital of Southern Medical University, Guangzhou, China; 7 Department of General Surgery, Nanfang Hospital, Southern Medical University, Guangzhou, China

## Abstract

**Importance:**

Occult peritoneal metastasis frequently occurs in patients with advanced gastric cancer and is poorly diagnosed with currently available tools. Because the presence of peritoneal metastasis precludes the possibility of curative surgery, there is an unmet need for a noninvasive approach to reliably identify patients with occult peritoneal metastasis.

**Objective:**

To assess the use of a deep learning model for predicting occult peritoneal metastasis based on preoperative computed tomography images.

**Design, Setting, and Participants:**

In this multicenter, retrospective cohort study, a deep convolutional neural network, the Peritoneal Metastasis Network (PMetNet), was trained to predict occult peritoneal metastasis based on preoperative computed tomography images. Data from a cohort of 1225 patients with gastric cancer who underwent surgery at Sun Yat-sen University Cancer Center (Guangzhou, China) were used for training purposes. To externally validate the model, data were collected from 2 independent cohorts comprising a total of 753 patients with gastric cancer who underwent surgery at Nanfang Hospital (Guangzhou, China) or the Third Affiliated Hospital of Southern Medical University (Guangzhou, China). The status of peritoneal metastasis for all patients was confirmed by pathological examination of pleural specimens obtained during surgery. Detailed clinicopathological data were collected for each patient. Data analysis was performed between September 1, 2019, and January 31, 2020.

**Main Outcomes and Measures:**

The area under the receiver operating characteristic curve (AUC) and decision curve were analyzed to evaluate performance in predicting occult peritoneal metastasis.

**Results:**

A total of 1978 patients (mean [SD] age, 56.0 [12.2] years; 1350 [68.3%] male) were included in the study. The PMetNet model achieved an AUC of 0.946 (95% CI, 0.927-0.965), with a sensitivity of 75.4% and a specificity of 92.9% in external validation cohort 1. In external validation cohort 2, the AUC was 0.920 (95% CI, 0.848-0.992), with a sensitivity of 87.5% and a specificity of 98.2%. The discrimination performance of PMetNet was substantially higher than conventional clinicopathological factors (AUC range, 0.51-0.63). In multivariable logistic regression analysis, PMetNet was an independent predictor of occult peritoneal metastasis.

**Conclusions and Relevance:**

The findings of this cohort study suggest that the PMetNet model can serve as a reliable noninvasive tool for early identification of patients with clinically occult peritoneal metastasis, which will inform individualized preoperative treatment decision-making and may avoid unnecessary surgery and complications. These results warrant further validation in prospective studies.

## Key Points

**Question:**

Can occult peritoneal metastasis be accurately assessed before surgery and without any invasive intervention?

**Findings:**

In this cohort study of 1978 patients, a deep neural network, the Peritoneal Metastasis Network, was developed for predicting occult peritoneal metastasis in gastric cancer based on preoperative computed tomography images. The model had excellent discrimination in external validation and substantially outperformed clinical factors.

**Meaning:**

The proposed deep learning model may be useful in preoperative treatment decision-making for avoiding unnecessary surgery and complications in certain patients.

## Teaser

This cohort study assesses the use of a deep learning model for predicting occult peritoneal metastasis based on preoperative computed tomographic images in patients with gastric cancer.

## Introduction

Gastric cancer (GC) is a common gastrointestinal malignant tumor and the second leading cause of cancer-related deaths worldwide.^1^ Peritoneal metastasis occurs in up to 66% of patients with advanced GC (AGC) and is considered an aggressive disease with poor outcomes.^2,3^ Patients with peritoneal metastasis are typically not eligible for curative surgery.^4^ Preoperative detection and diagnosis of peritoneal metastasis are therefore critical to inform treatment decision-making and avoid unnecessary surgery.

Computed tomography (CT) is the most widely used imaging modality for diagnosis of peritoneal metastasis. Certain CT features, such as omental cake, large ascites, and peritoneal thickening, may be used to diagnose the presence of peritoneal metastasis. However, these classic signs typically appear at a late stage, and tumor implants less than 1 cm are often missed, resulting in a low sensitivity of 28.3% to 50.9%.^5,6^ These peritoneal metastases undetected by CT but later diagnosed during exploratory laparoscopy or laparotomy are considered clinically occult peritoneal metastasis. However, because of cost-effectiveness concerns, routine staging laparoscopy is not being universally used, especially in East Asia.^7,8^ Therefore, an unmet need remains for accurate and sensitive prediction of occult peritoneal metastasis using a noninvasive means.

Deep learning, in particular, convolutional neural networks, has emerged as a powerful technique for extracting subtle information from medical images.^9,10,11^ These techniques have demonstrated excellent diagnostic performance in a variety of clinical applications.^9,10,11,12,13,14^ Recently, deep learning has been used to predict response and survival outcomes after adjuvant chemotherapy in GC.^11^ We developed a deep learning model, the Peritoneal Metastasis Network (PMetNet), to predict clinically occult peritoneal metastasis using preoperative CT in patients with GC.

## Methods

### Patients

In this multicenter, retrospective cohort study, we collected clinicopathological and image data from 1978 patients with AGC who were consecutively treated at 3 hospitals (Table 1). The training cohort comprised 1225 consecutive patients who underwent operation at Sun Yat-sen University Cancer Center (Guangzhou, China) between January 1, 2008, and January 31, 2013. The external validation cohorts comprised 504 patients who underwent operation at Nanfang Hospital (Guangzhou, China) between January 1, 2007, and December 31, 2017, with the same enrollment criteria and 249 patients from the Third Affiliated Hospital of Southern Medical University (Guangzhou, China) between January 1, 2007, and December 31, 2017, with the same enrollment criteria. This study followed the Transparent Reporting of a Multivariable Prediction Model for Individual Prognosis or Diagnosis (TRIPOD) reporting guideline. The institutional review board at each participating center (Sun Yat-sen University Cancer Center, Nanfang Hospital, and the Third Affiliated Hospital of Southern Medical University, Guangzhou, Guangdong, People's Republic of China) approved this study. Patient informed consent was waived given the retrospective design, and all data were deidentified.

**Table 1.** Characteristics of Patients With Gastric Cancer in Training and Validation Cohorts^a^

Characteristic	Training cohort (n = 1225)	External validation		*P* value
		Cohort 1 (n = 504)	Cohort 2 (n = 249)	
Sex				
Female	384 (31.3)	164 (32.5)	80 (32.1)	.60
Male	841 (68.7)	340 (67.5)	169 (67.9)	
Age, mean (SD), y	56.5 (11.9)	54.8 (12.4)	55.6 (12.6)	.02
Age, y				
<60	696 (56.8)	303 (60.1)	102 (41.0)	.12
≥60	529 (43.2)	201 (39.9)	147 (59.0)	
Major location				
Cardia	414 (33.8)	99 (19.6)	63 (25.3)	<.001
Body	250 (20.4)	103 (20.4)	45 (18.1)	
Antrum	502 (41.0)	274 (54.4)	137 (55.0)	
Whole	59 (4.8)	28 (5.6)	4 (1.6)	
Biopsy differentiation				
Well or moderate	216 (17.6)	169 (33.5)	93 (37.3)	<.001
Poor	1009 (82.4)	335 (66.5)	156 (62.7)	
Lauren type				
Intestinal or mixed	784 (64.0)	351 (69.6)	176 (70.7)	<.001
Diffuse	441 (36.0)	153 (30.4)	73 (29.3)	
CEA				
Normal	986 (80.5)	54 (10.7)	24 (9.6)	<.001
Elevated	239 (19.5)	450 (89.3)	225 (90.4)	
CA19-9				
Normal	986 (80.5)	65 (12.90)	34 (13.7)	<.001
Elevated	239 (19.5)	439 (87.10)	215 (86.3)	
Clinical T stage				
T2/T3	332 (27.1)	149 (29.6)	103 (41.4)	<.001
T4	893 (72.9)	355 (70.4)	146 (58.6)	
Clinical N stage				
N0	435 (35.5)	214 (42.5)	91 (36.5)	.02
N+	790 (64.5)	290 (57.5)	158 (63.5)	

Abbreviations: CA19-9, cancer antigen 19-9; CEA, carcinoembryonic antigen; N0, without lymph node metastasis; N+, with lymph node metastasis.

^a^ Data are presented as number (percentage) of patients unless otherwise indicated.

All patients received open (1048 [53%]) or laparoscopic (930 [47%]) surgery. The inclusion criteria were histologically confirmed GC, standard unenhanced and contrast-enhanced abdominal CT performed less than 30 days before operation, complete clinicopathological data, no combined malignant neoplasm, and CT images obtained before operation. We excluded 273 patients who had stage T1 tumors given a low risk of peritoneal metastasis in early-stage disease or whose primary tumor could not be identified at CT as well as patients who had received previous anticancer therapy before surgery (such as neoadjuvant chemotherapy).

Preoperative staging for most patients enrolled in this study was based on CT images, and only a small proportion of patients (<10%) underwent endoscopic ultrasonography, positron emission tomography, or staging laparoscopy before surgery. All patients’ peritoneal metastasis statuses were initially diagnosed as negative by a radiologist’s interpretation of the CT images. During surgery, the patient’s peritoneum was carefully evaluated by the surgeons. For patients who had peritoneal metastasis suggestive of cancer, samples of their peritoneal implants or ascites were sent for pathological or cytological examination. The presence of peritoneal metastasis was identified according to the American Joint Committee on Cancer guidelines by consensus between the pathologists and surgeons.

Baseline clinicopathological data, including age, sex, preoperative differentiation status, Lauren type, carcinoembryonic antigen, and cancer antigen 19-9, were collected from medical records. The clinical characteristics of the 1978 patients are listed in Table 1. Data analysis was performed between September 1, 2019, and January 31, 2020.

### Image Acquisition and Processing

All patients underwent contrast-enhanced abdominal CT before surgery. Portal venous-phase CT images were retrieved from the Picture Archiving and Communication System (Carestream). Details regarding the CT acquisition parameters and image retrieval procedure are presented in the eMethods in the Supplement. The primary tumor was manually delineated on the CT images by 2 radiologists (C.C. and Q.Y.) by consensus (with 11 and 10 years of clinical experience in abdominal CT interpretation, respectively) using the ITK-SNAP software.^15^ Both radiologists (C.C. and Q.Y.) were blinded to the clinical and histopathological data but were aware that the patients had GC. Any discrepancy was resolved by a third radiologist (Y.X., with 31 years of experience in abdominal CT interpretation). The reason for including tumor delineation was to ensure that the deep learning network’s attention was focused on the most relevant part of the image for prediction purposes. This network has been shown to outperform networks that are trained without the tumor mask.^11^

### Development of the Deep Learning Model

We proposed a densely connected convolutional network combined with long-short connections (DCCN-LSC) to predict peritoneal metastasis based on CT image (eFigure 1 in the Supplement). The DCCN-LSC model (eFigure 1A in the Supplement) consists of a convolutional layer, 2 dense blocks each followed by a transition layer, and a final dense block followed by a pooling and a linear layer. The dense blocks (eFigure 1B in the Supplement) use short dense connectivity among sequences of convolution, batch normalization, and rectified linear units. The transition layers, formed by a convolutional and a pooling layer, are used to reduce the dimension of the feature maps between adjacent dense blocks. The final pooling and linear layers are used to reduce the output dimension to peritoneal metastasis prediction. All convolutional operators use a stride of 2 and a kernel size of 3. Different from a traditional dense network, DCCN-LSC introduces a long connection that enables the model to extract multilevel features of the tumor, which are incorporated into the final fully connected layer for peritoneal metastasis prediction (eFigure 2 in the Supplement). The input data to the network are an image patch of 10 × 10 cm^2^ in the CT slice that contains the largest area of the primary tumor along with its segmentation mask. The network output is the probability of occult peritoneal metastasis.

**Figure 1.**
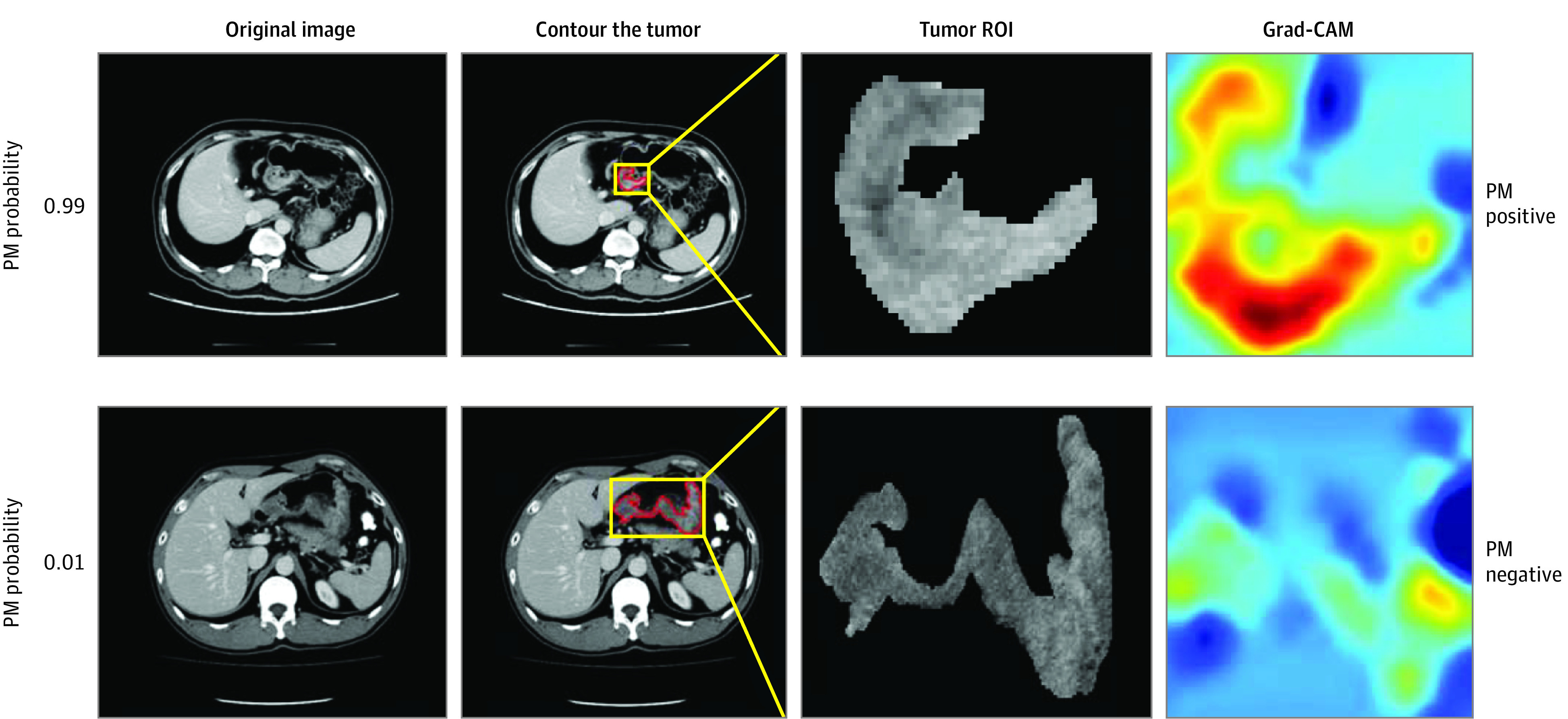
Two Representative Computed Tomography (CT) Images and the Corresponding Output From the Deep Learning Model The gradient-weighted class activation mapping (Grad-CAM) was used to highlight which areas of the CT image are important in generating a particular prediction. The numbers 0.99 and 0.01 represent the predicted probability of peritoneal metastasis (PM). ROI indicates region of interest.

For training purposes, we used several strategies to minimize overfitting, including cross-validation, data augmentation, batch normalization, and early stopping. To visualize which areas of the CT image are important in generating a particular prediction, we introduce guided gradient-weighted class activation mapping (Grad-CAM) (Figure 1). The code for the implementation of networks is publicly available at GitHub.^16^ For a detailed description of the network architecture and training process, please refer to the eMethods in the Supplement.

### Performance Evaluation and Comparison With Clinicopathological Factors

We assessed the associations between clinical characteristics and peritoneal metastasis using univariate analysis. Multivariable logistic regression was applied to select independent predictors of peritoneal metastasis. Variables that achieved a predefined statistical significance at *P* < .05 were selected as independent predictors in the multivariable model. We then built a nomogram with the combined PMetNet signature and clinical features based on the multivariable logistic regression. The prediction model was validated by measuring the discrimination and calibration using bootstrapping with 1000 times of resampling. Calibration plots were used to graphically represent the agreement between the predicted and actual probability of occult peritoneal metastasis. A decision curve analysis was performed to evaluate the model’s clinical usefulness by quantifying the net benefit at various threshold probabilities.^17^

### Statistical Analysis

Differences in distributions between the variables examined were assessed with the unpaired, 2-tailed χ^2^ test or the Fisher exact test, as appropriate. The area under the receiver operating characteristic curve (AUC) was also used to measure discriminative ability of the prediction models. Nomograms and calibration plots were generated using the rms package of R software, version 3.5.1 (R Foundation for Statistical Computing). Statistical analyses were performed with R software, version 3.5.1 and SPSS, version 21.0 (IBM Corp). All statistical tests were 2 sided with a significance level of *P* < .05.

## Results

### Clinical Characteristics

A total of 1978 patients (mean [SD] age, 56.0 [12.2] years; 1350 [68.3%] male) were included in the study. The clinicopathological characteristics for the training cohort (n = 1225), external validation cohort 1 (n = 504), and external validation cohort 2 (n = 249) are listed in Table 1. A significant difference was found between existing occult peritoneal metastasis and no peritoneal metastasis in the lesion location, Lauren type, and biopsy differentiation degree (eTable in the Supplement).

### Prediction Performance of the Deep Learning Model

**Figure 2.**
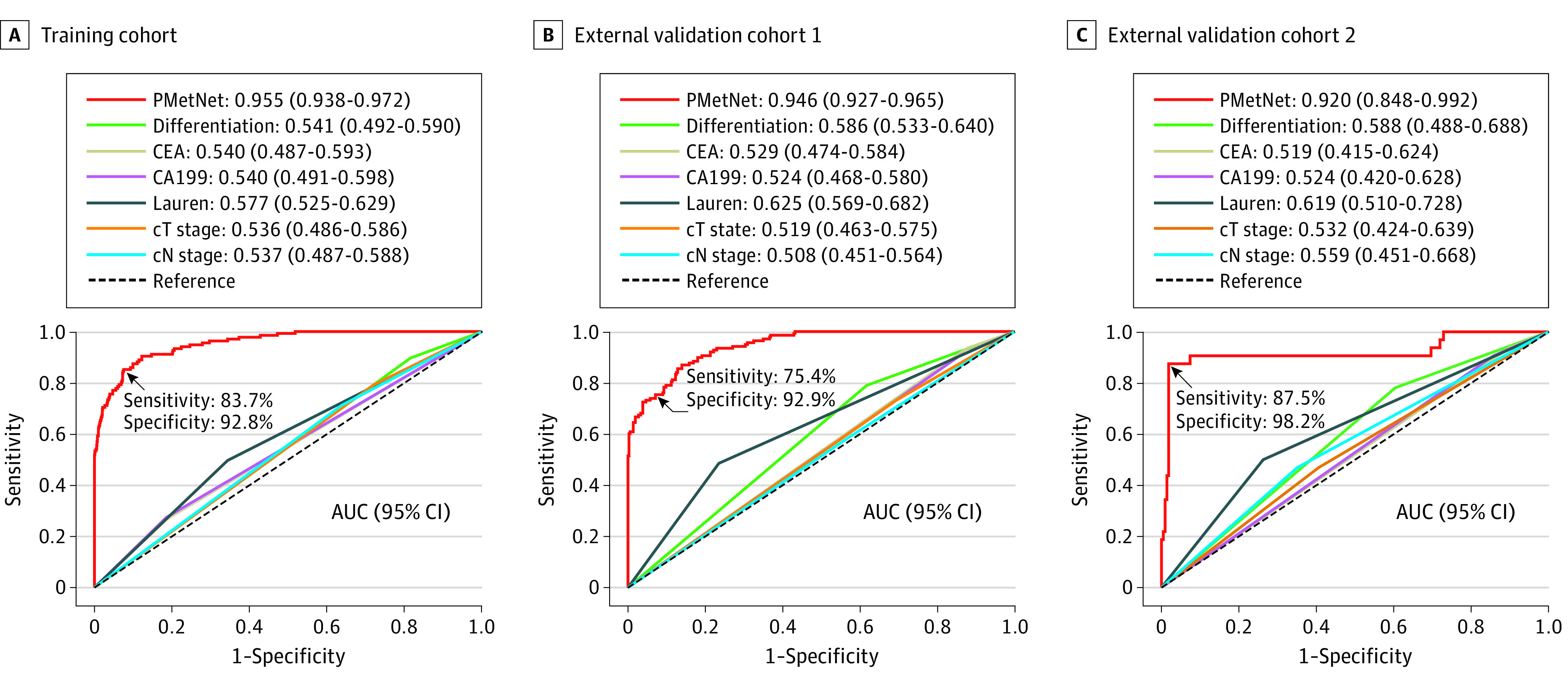
Area Under the Receiver Operating Characteristic Curves (AUCs) Derived From the Peritoneal Metastasis Network (PMetNet) and Clinicopathological Factors for the Diagnosis of Occult Peritoneal Metastasis in the Training and 2 Validation Cohorts CA199 indicates cancer antigen 19-9; CEA, carcinoembryonic antigen.

We proposed a novel end-to-end deep neural network, PMetNet, to predict occult peritoneal metastasis of a patient based on CT image. The deep learning model, PMetNet, accurately predicted occult peritoneal metastasis in the training cohort. Receiver operating characteristic curve analysis showed that the AUC was 0.955 (95% CI, 0.938-0.972). The model achieved a sensitivity of 83.7% at a high specificity of 92.8% for predicting the presence of peritoneal metastasis (Figure 2).

For validation, the PMetNet model had a similar prediction performance in the 2 external validation cohorts, with AUCs of 0.946 (95% CI, 0.927-0.965) in external validation cohort 1 and 0.920 (95% CI, 0.848-0.992) in external validation cohort 2. With the same cutoff, the model showed sensitivities of 75.4% and 87.5% and specificities of 92.9% and 98.2% in the 2 external validation cohorts, respectively. Of note, the model had high negative predictive values of 97.9% in the training cohort, 90.9% in external validation cohort 1, and 98.2% in external validation cohort 2 (Table 2).

**Table 2.** Diagnostic Performance of the Peritoneal Metastasis Network for the Assessment of Peritoneal Metastasis in the Training and Validation Cohorts

Cohort	AUC (95% CI)	Sensitivity (95% CI)	Specificity (95% CI)	PPV (95% CI)	NPV (95% CI)
Training cohort	0.955 (0.938-0.972)	83.7 (76.4-89.5)	92.75 (91.0-94.2)	58.9 (51.5-65.9)	97.9 (96.8-98.7)
External validation					
Cohort 1	0.946 (0.927-0.965)	75.4 (67.3-82.3)	92.9 (89.8-95.3)	80.0 (72.1-86.5)	90.9 (87.5-93.6)
Cohort 2	0.920 (0.848-0.992)	87.5 (71.0-96.5)	98.2 (95.3-99.5)	87.5 (71.0-96.5)	98.2 (95.3-99.5)

Abbreviations: AUC, area under the receiver operating characteristic curve; NPV, negative predictive value; PPV, positive predictive value.

### Comparison and Integration With Clinicopathological Variables

The discrimination performance of PMetNet was substantially higher than conventional clinicopathological variables in all 3 cohorts (AUC range, 0.51-0.63) (Figure 2). On multivariable analysis, PMetNet, Lauren type, and tumor differentiation were significant predictors of peritoneal metastasis (Table 3). We then combined these variables and built a nomogram (eFigure 3 in the Supplement), which yielded slightly higher accuracy than PMetNet, with AUCs of 0.950 (95% CI, 0.932-0.968) and 0.953 (95% CI, 0.914-0.992) in the 2 validation cohorts. The calibration curve also showed excellent agreement between the predicted and observed probabilities for occult peritoneal metastasis in all cohorts (eFigure 4 in the Supplement). However, these differences were small and not statistically significant, suggesting that the PMetNet model played a dominant role in predicting occult peritoneal metastasis (eFigure 5 in the Supplement).

**Table 3.** Multivariate Logistic Regression Analysis for the Diagnosis of Peritoneal Metastasis in Patients With Gastric Cancer in Each Cohort

Variable	OR (95% CI)	*P* value
**Training cohort**		
PMetNet (>0.5 vs <0.5)	63.77 (37.88-107.33)	<.001
Lauren type (diffuse vs intestinal or mixed)	3.511 (1.561-7.898)	.002
Differentiation (poor vs moderate and well)	2.915 (1.668-5.092)	<.001
**External validation cohort 1**		
PMetNet (>0.5 vs <0.5)	46.77 (24.74-88.42)	<.001
Lauren type (diffuse vs intestinal or mixed)	2.522 (1.728-3.681)	<.001
Differentiation (poor vs moderate and well)	3.129 (1.690-5.792)	<.001
**External validation cohort 2**		
PMetNet (>0.5 vs <0.5)	481.7 (91.37-2539)	<.001
Lauren type (diffuse vs intestinal or mixed)	5.172 (0.967-27.66)	.055

Abbreviations: OR, odds ratio; PMetNet, Peritoneal Metastasis Network.

The decision curve analysis showed that, for a wide range of threshold probabilities from 5% to 75%, using the PMetNet to predict occult peritoneal metastasis provides additional clinical benefit compared with the intervention-for-all and intervention-for-none strategy as well as common clinicopathological variables (eFigure 5 in the Supplement).

## Discussion

To our knowledge, this cohort study is the first and largest (nearly 2000 patients) study to use deep learning for the prediction of occult peritoneal metastasis in patients with GC. Peritoneal metastasis is a major pattern of metastatic spread in GC and represents an incurable disease with a poor prognosis.^2,3^ The presence of peritoneal metastasis precludes the treatment option of curative surgery. Therefore, it is crucial to reliably identify patients with peritoneal metastasis to spare them from unnecessary extensive gastrectomy, which can involve serious complications, such as bleeding and infection.^18,19^ Such tools are currently lacking in a preoperative setting. The current study addressed this unmet need by applying deep learning methods to extract subtle information from preoperative CT images. In multi-institutional validation, this model substantially improved diagnostic accuracy for predicting the presence of occult peritoneal metastasis. Given a much higher sensitivity (75%-88% vs 28%-51% in previous reports^5,6^), such a model could be used to identify more patients with clinically occult peritoneal metastasis, which might otherwise be missed by the radiologist.

Several recent studies^20,21,22^ have developed radiomics-based signatures to predict peritoneal metastasis. However, this approach relies on handcrafted definition of specific features, which is biased by developer experience and may miss important information contained in the image. Compared with a recent radiomics study,^20^ the model in this study achieved consistently better diagnostic performance with higher AUCs, sensitivity, and specificity, confirming the power of the deep learning approach. Importantly, this study included only patients with clinically occult peritoneal metastasis. The sensitivity of PMetNet may be higher when considering all patients with peritoneal metastasis.^6^

To gain insight into how the deep learning network produced an output, the study used Grad-CAM to provide visualization of what regions were highlighted on the original CT images. By examining the network visualization for thousands of patients, the class activation map was found not to be uniform and only certain intratumoral regions were activated (Figure 1). This finding suggests that intratumoral heterogeneity may be an important factor for determining peritoneal metastasis. Indeed, imaging heterogeneity of tumor phenotypes has been associated with aggressive biology and poor prognosis in many cancers.^23,24^ Taken together, these results demonstrate that deep learning based on CT images can be used to uncover subtle relations between tumor characteristics and metastatic potential.

This study found that among traditional clinical risk factors, the only consistent predictor of peritoneal metastasis across 3 cohorts was Lauren type. However, combining clinical variables with the imaging model did not significantly improve the results over imaging alone. This finding suggests that the risk of occult peritoneal metastasis is mainly contained in the primary tumor. This suggestion reaffirms the approach of incorporating the tumor mask into the deep learning network, which allows its attention to focus on the most relevant region for prediction.

The current study is focused on patients who underwent resection but did not receive any systemic therapies before surgery. In future work, it will be interesting to test whether the proposed deep learning model will identify peritoneal disease in patients who have been treated with neoadjuvant chemotherapy, which is the standard treatment for local AGC in the US. In addition, imaging may be combined with other complementary data, such as blood and serum biomarkers, to further enhance the prediction accuracy.

### Limitations

This study has limitations, the main one being its retrospective nature, although the study included patients from 3 different institutions to assess its reproducibility. The study was focused on patients with GC in Asian populations, and differences exist in the prevalence and presentation of disease for patients in Western countries, whose cancers tend to be diagnosed at more advanced disease stages. Therefore, the model’s performance in different ethnic groups should be further evaluated. In addition, CT images were collected from several scanners, and acquisition parameters varied. The model’s generalizability across diverse populations and scanners should be rigorously tested. Because the network requires tumor contour with manual delineation, robust automated segmentation tools should be developed to reduce interobserver variation. Given the low image contrast and extensive anatomical variations in GC, fully automated tumor segmentation with high accuracy and consistency remains a challenging task, even with state-of-the-art deep learning approaches. Further investigation and development of more robust segmentation methods are needed. The current model is based on 2-dimensional imaging as an input. Future models incorporating 3-dimensional imaging might further improve performance.

Finally, the deep learning model’s specificity is not perfect, with a false-positive rate of 2% to 7%. Currently, this model cannot be used alone to exclude patients who might be eligible for curative surgery. For clinical applications, it will be crucial to integrate information from investigations of other modalities, such as endoscopic ultrasonography and/or laparoscopy, to improve the specificity and sensitivity for the diagnosis of occult peritoneal metastasis.

## Conclusions

These findings represent an important step forward in the accurate prediction of occult peritoneal metastasis by applying advanced deep learning techniques. If validated in prospective studies, the PMetNet model could serve as a useful approach for the noninvasive prediction of occult peritoneal metastasis and may inform individualized surgical management of GC.

## Notes

Supplement. **eMethods.** Supplementary Methods **eReferences.** **eFigure 1.** Proposed Densely Connected Convolutional Networks Combined With Long-Short Connections (DCCN-LSC) for Occult Peritoneal Metastasis Prediction **eFigure 2.** Plots Showing the Performance of the DCCN-LSC Algorithm in Training Set With an Increase in Each Epoch **eFigure 3.** The Combined Nomogram for Peritoneal Metastasis Prediction **eFigure 4.** Decision Curve Analysis for the Nomogram, PMetNet and Clinicopathological Factors in the Training and External Validation Cohorts **eFigure 5.** Calibration Curves of the Nomogram in the Training Cohort and Validation Cohorts **eTable.** Characteristics of Patients With Gastric Cancer in Training and Validation Cohorts
