# Supplementary material for: Noninvasive Prediction of Occult Peritoneal Metastasis in Gastric Cancer Using Deep Learning
Source: JAMA Netw Open. 2021 Jan 5;4(1):e2032269. doi: 10.1001/jamanetworkopen.2020.32269 (PMC7786251; doi:10.1001/jamanetworkopen.2020.32269)
Supplement: Supplement. — eMethods. Supplementary Methods eReferences. eFigure 1. Proposed Densely Connected Convolutional Networks Combined With Long-Short Connections (DCCN-LSC) for Occult Peritoneal Metastasis Prediction eFigure 2. Plots Showing the Performance of the DCCN-LSC Algorithm in Training Set With an Increase in Each Epoch eFigure 3. The Combined Nomogram for Peritoneal Metastasis Prediction eFigure 4. Decision Curve Analysis for the Nomogram, PMetNet and Clinicopathological Factors in the Training and External Validation Cohorts eFigure 5. Calibration Curves of the Nomogram in the Training Cohort and Validation Cohorts eTable. Characteristics of Patients With Gastric Cancer in Training and Validation Cohorts [file jamanetwopen-e2032269-s001.pdf]

Supplementary Online Content

Jiang Y, Liang X, Wang W, et al. Noninvasive prediction of occult peritoneal metastasis in gastric cancer using deep learning. *JAMA Netw Open*. 2021;4(1):e2032269. doi:10.1001/jamanetworkopen.2020.32269

eMethods. Supplementary Methods

eReferences.

eFigure 1. Proposed Densely Connected Convolutional Networks Combined With Long-Short Connections (DCCN-LSC) for Occult Peritoneal Metastasis Prediction

eFigure 2. Plots Showing the Performance of the DCCN-LSC Algorithm in Training Set With an Increase in Each Epoch

eFigure 3. The Combined Nomogram for Peritoneal Metastasis Prediction

eFigure 4. Decision Curve Analysis for the Nomogram, PMetNet and Clinicopathological Factors in the Training and External Validation Cohorts

eFigure 5. Calibration Curves of the Nomogram in the Training Cohort and Validation Cohorts

eTable. Characteristics of Patients With Gastric Cancer in Training and Validation Cohorts

This supplementary material has been provided by the authors to give readers additional information about their work.

© 2021 Jiang Y et al. *JAMA Network Open.*

eMethods. Supplementary Methods

1. CT Acquisition and Image Processing

All patients underwent contrast-enhanced abdominal CT using the multidetector row CT (MDCT) systems (GE Lightspeed 16, GE Healthcare Milwaukee, WI; 64-section LightSpeed VCT, GE Medical Systems, Milwaukee, WI; USA). Following intravenous contrast administration, arterial and portal venous-phase contrast-enhanced CT scans were performed after delays of 28 s and 60 s, respectively. Iodinated contrast material in the amount of 90 - 100 ml (Ultravist 370, Bayer Schering Pharma, Berlin, Germany) was injected at a rate of 3.0 or 3.5 ml/s with a pump injector (Ulrich CT Plus 150, Ulrich Medical, Ulm, Germany). The CT acquisition protocols were as follows: 120 kV; 150-190 mAs; 0.5- or 0.4-second rotation time. Contrast-enhanced CT was reconstructed with a field of view, 350×350 mm; data matrix, 512×512; in-plane spatial resolution 0.607-0.751 mm; axial slice thickness 5.0 mm for 98% patients with a range of 1.25-7.5 mm.

We analyzed the portal venous-phase CT images because of well differentiation between the tumor tissue and adjacent normal bowel wall. The relatively coarse and heterogeneous resolution in z-axis compared with in-plane resolution would not allow a meaningful and reliable 3D analysis of the image. Therefore, we focused on the most representative 2D slice, i.e., largest tumor section in the axial plane. Two radiologists C.C. and Q.Y. (with 11 and 10 years of clinical experience in abdominal CT interpretation, respectively) manually delineated the primary tumor on the CT images by using the ITK-SNAP (http://www.itksnap.org)[1, 2].

© 2021 Jiang Y et al. *JAMA Network Open.*

2. Development of Deep Learning Signature

2.1 Network architecture

The proposed DCCN-LSC deep learning model (Figure 1A) consists of a convolutional layer, two dense blocks each followed by a transition layer, and a final dense block followed by a pooling and a linear layer. The dense blocks (Figure 1B) use short dense connectivity between sequences of convolution, batch normalization, and rectified linear units (ReLU). The transition layers, formed by a convolutional and a pooling layer, are used to reduce the dimension of the feature maps between adjacent dense blocks. The final pooling and linear layers are used to reduce the output dimension to PM prediction. All convolutional operators use a stride of 2 and the kernel size of 3. After the CT image input, we add a convolutional layer with the 2×2×2 stride. The convolutional layer is followed by four dense blocks, which use dense connectivity formed by the output feature from all the prior layers:

$$x_l = H_l([x_0, x_1, \dots, x_{l-1}])$$

where $[x_0, x_1, \dots, x_{l-1}]$ is the tensor that concatenating the feature maps from all previous layers. H_l is a non-linear transformation function of three sequential processes: convolution, batch normalization, and rectified linear units. We use the shortcut connection to enable the dense layer can receive the feature maps from all the previous dense layers. We set a transition layer to reduce the dimension of the feature maps between the adjacent dense blocks. The transition layer is formed by a convolutional and pooling layer. To make a regression to the occult PM prediction, we add a pooling and a linear layer to the last dense block for reducing the dimension of

the feature map.

Different from traditional dense-net that only with the short connection inside the dense blocks, DCCN-LSC introduces a long connection that enables the model to extract the multi-level feature of the tumor. The multi-level feature maps are incorporated into the final fully connection layer for accurate occult PM prediction.

2.2 Data augmentation

As a general problem in the deep network training [4], the limited training data post a big challenge in occult PM prediction with the CT image. In this section, we try to deal with this problem using the image augmentation, which is usually implemented in deep neural network [5]. For taking into account the different positions of the tumor in the CT image, the proposed augmentation model first applies the random geometric transformation to the CT image. Different from the conventional augmentation technique, to improve the accuracy of occult PM prediction, we introduce random image transformation processing to imitate the image acquired from different machines and different hospitals. The random image transformation does not require any parameter learning, which can be easily implemented to the other convolutional neural network (CNN) regression tasks. The details of the random geometric and image transformation are explained as follows.

The geometric transformations include rotation, translation, rescaling, and deformation. The rotation angles are randomly chosen from -10° to 10°. The translation distances are randomly generated from -50 mm to 50 mm. The scaling factor is

© 2021 Jiang Y et al. *JAMA Network Open.*

randomly from 0.9 to 1.1. To simulate the tumor deformation, the CT is distorted into the other patients' CT images to generate the deformable image using the image registration method. The selected parameter here is reflected in the possible geometric changes.

The image transformation includes random Gaussian filtering and noise. The image transformations are managed with a certain probability. Given the image in a mini-batch, the probability of this image being implemented random image transformation is p. Gaussian filtering and the noise selects a standard deviation of the Gaussian distribution randomly in the range specified by the minimum and the maximum.

2.3 Implementation detail

The loss function of the occult PM prediction is binary cross-entropy. To minimize the loss function, we use Adam algorithm to obtain the optimal parameters. The learning rate is set at 10^{-2} initially and then gradually decreased slowly decrease it to 10^{-6}. The DCNN-LSC model was trained for 100 epochs with a batch size of 16. We train the data using Matlab on 4 NVIDIA GeForce GTX 1080 Ti GPUs, an Intel Xeon(R) CPU E5-1650 v4 @ 3.60GHz × 12, and 64 GB of internal memory.

eReferences

1. Jiang Y, Yuan Q, Lv W, et al. Radiomic signature of (18)F fluorodeoxyglucose PET/CT for prediction of gastric cancer survival and chemotherapeutic benefits. *Theranostics* 2018; 8(21):5915-5928.

© 2021 Jiang Y et al. *JAMA Network Open.*

2. Yushkevich PA, Piven J, Hazlett HC, et al. User-guided 3D active contour segmentation of anatomical structures: Significantly improved efficiency and reliability. *Neuroimage* 2006; 31(3):1116-1128.

3. Huang G, Liu Z, Van Der Maaten L, et al. Densely connected convolutional networks. Proceedings of the IEEE conference on computer vision and pattern recognition, 2017. pp. 4700-4708.

4. LeCun Y, Bengio Y, Hinton G. Deep learning. *Nature* 2015; 521(7553):436-44.

5. Bloice MD, Stocker C, Holzinger A. Augmentor: an image augmentation library for machine learning. *arXiv preprint arXiv:1708.04680* 2017.

© 2021 Jiang Y et al. *JAMA Network Open.*

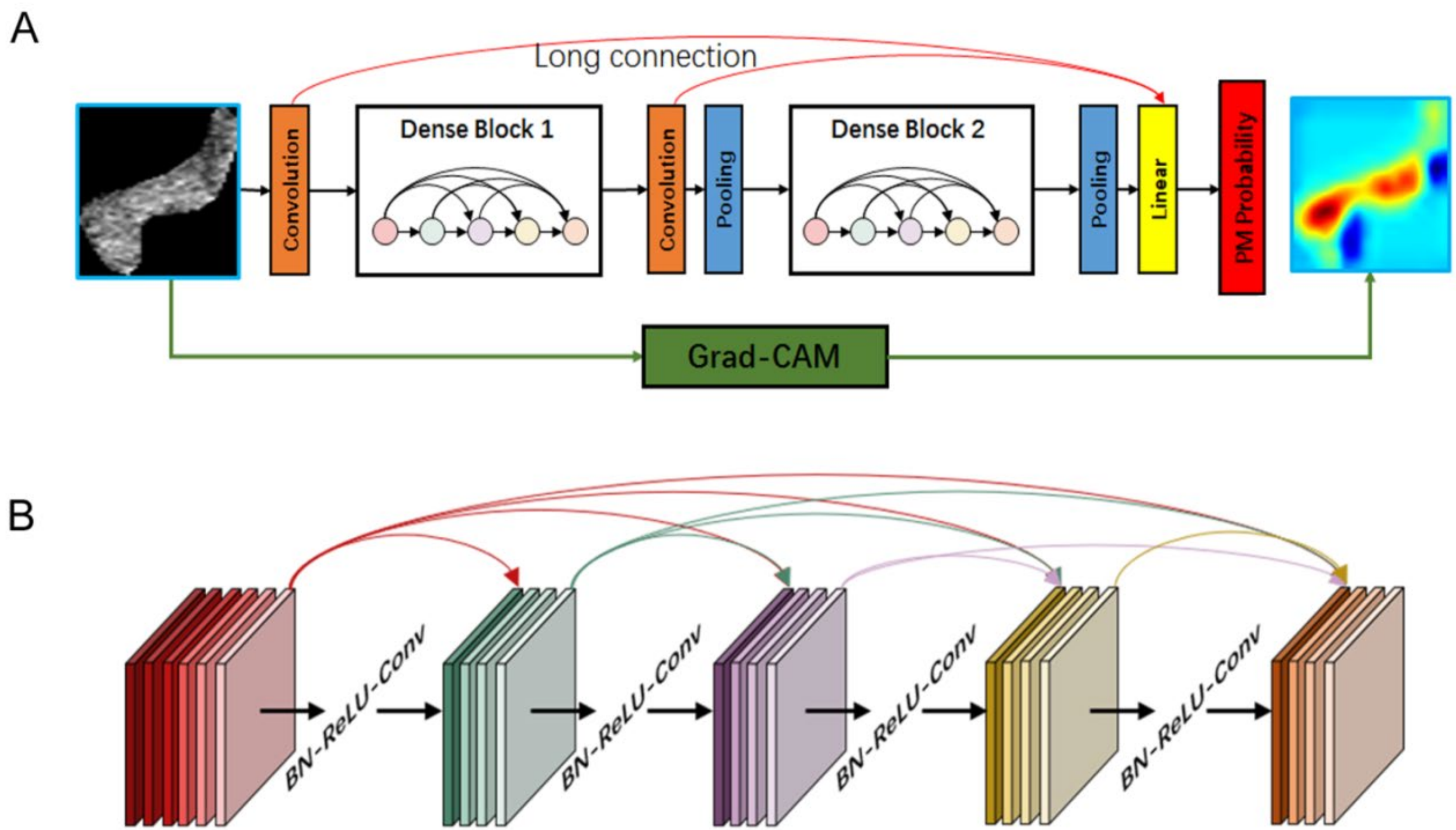

eFigure 1. Proposed Densely Connected Convolutional Networks combined with Long-Short Connections (DCCN-LSC) for occult PM prediction.

(A) Schematic of the network. (B) Architecture of the underlying dense block.

© 2021 Jiang Y et al. *JAMA Network Open.*

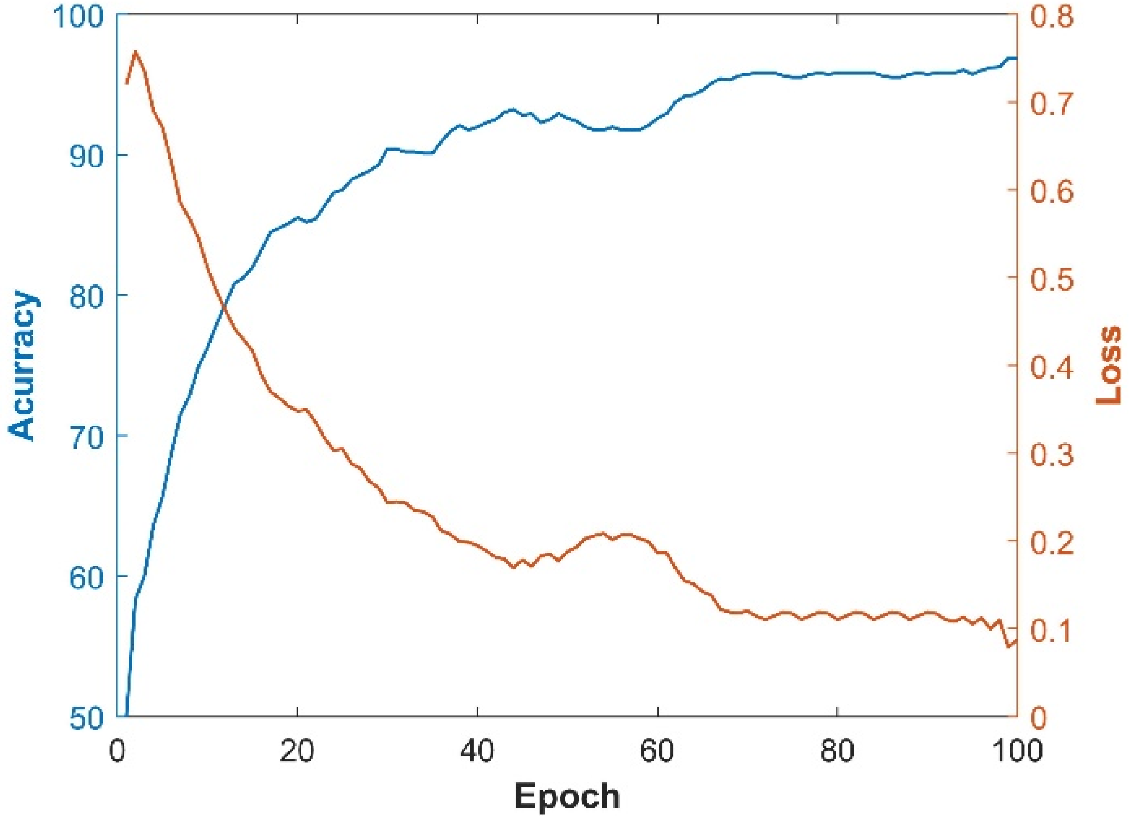

eFigure 2. Plots showing the performance of the DCCN-LSC algorithm in training set with an increase in each epoch

Accuracy and cross-entropy loss are plotted against the training epoch.

© 2021 Jiang Y et al. *JAMA Network Open.*

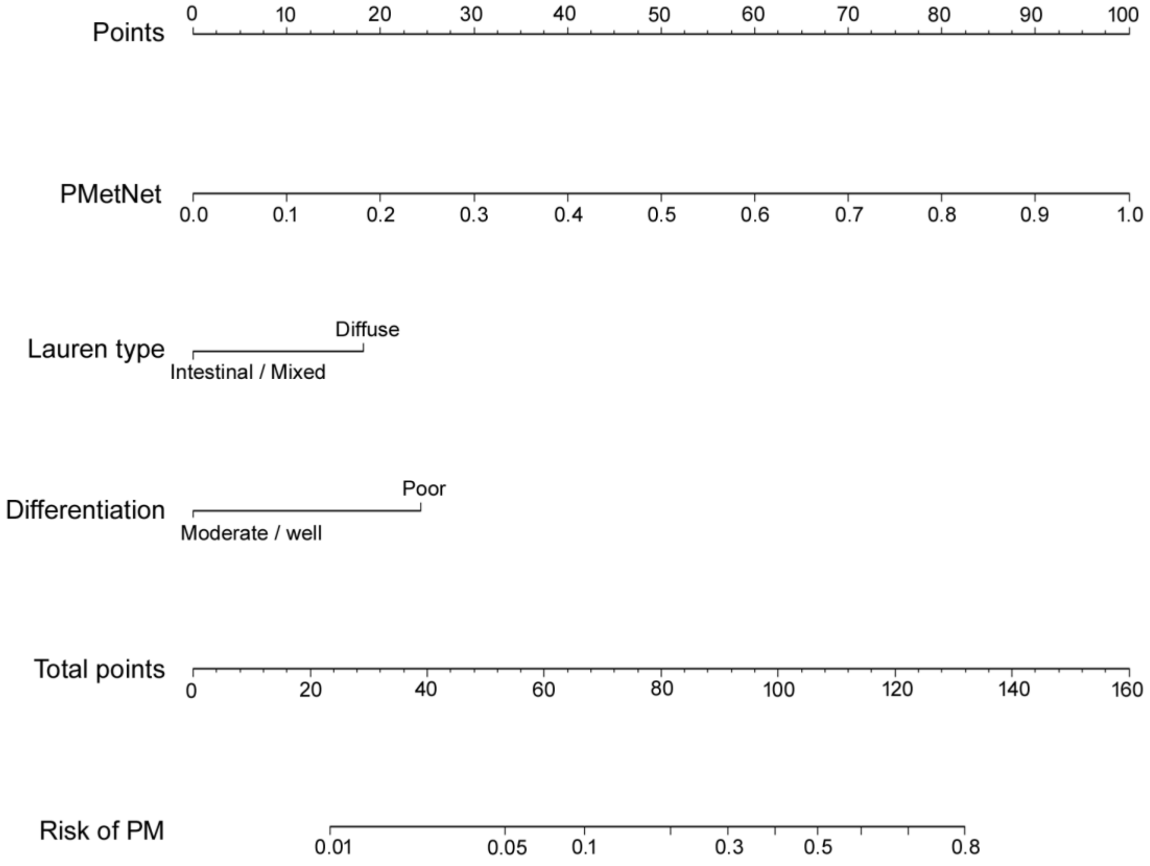

eFigure 3. The combined nomogram for PM prediction

PM, peritoneal metastasis.

© 2021 Jiang Y et al. *JAMA Network Open.*

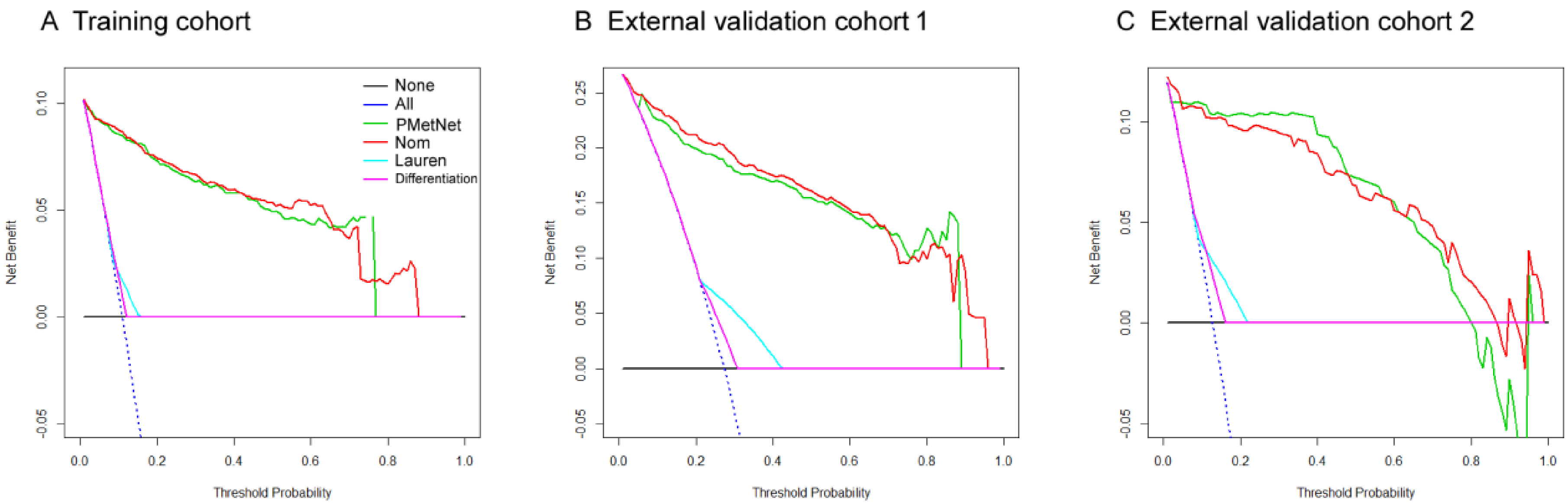

eFigure 4. Decision curve analysis for the nomogram, PMetNet and clinicopathological factors in the training and external validation cohorts

© 2021 Jiang Y et al. *JAMA Network Open.*

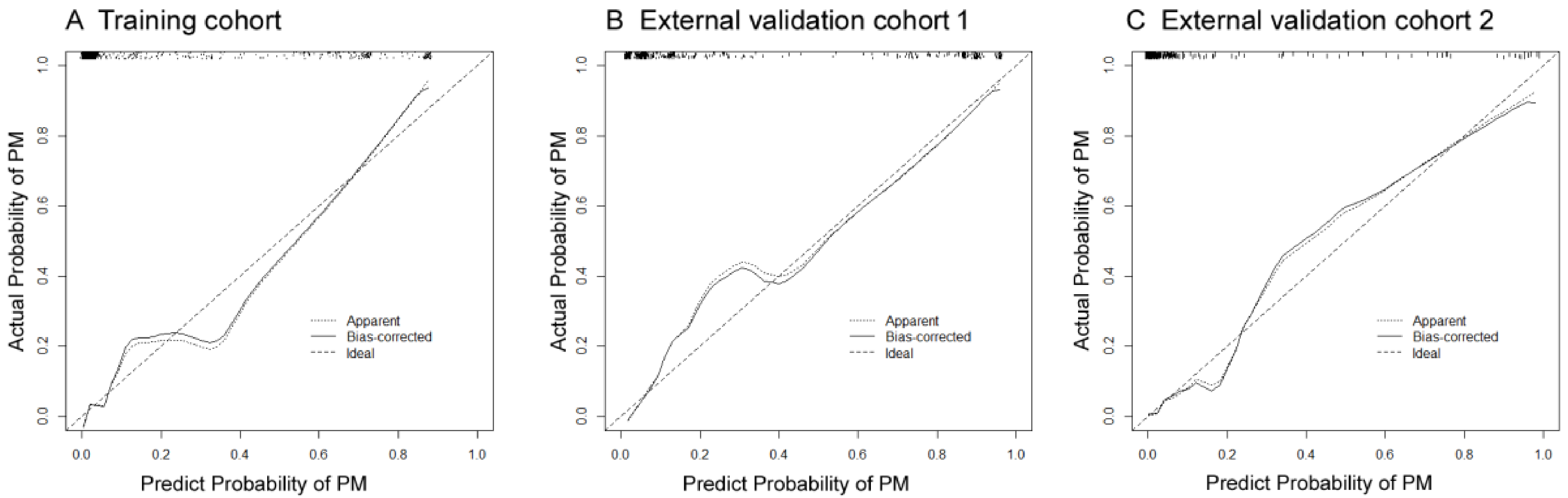

eFigure 5. Calibration curves of the nomogram in the training cohort and validation cohorts

© 2021 Jiang Y et al. *JAMA Network Open.*

eTable. Characteristics of Patients With Gastric Cancer in Training and Validation Cohorts

Variables	Training cohort (n = 1225)					External validation cohort 1 (n = 504)					External validation cohort 2 (n = 249)				
	PM-		PM+		*P*	PM-		PM+		*P*	PM-		PM+		*P*
**Gender**					0.357					0.405					0.603
Female	337	30.9%	47	34.8%		123	33.6%	41	29.7%		71	32.7%	9	28.1%	
Male	753	69.1%	88	65.2%		243	66.4%	97	70.3%		146	67.3%	23	71.9%	
**Age(years), median(IQR)**	58(50-65)		57(46-63)			56(47-64)		56(50-64)			57(48-65)		53(49-62)		
**Age(years)**					0.672					0.224					0.231
＜60	617	56.6%	79	58.5%		226	61.7%	77	55.8%		92	42.4%	10	31.3%	
≧60	473	43.4%	56	41.5%		140	38.3%	61	44.2%		125	57.6%	22	68.8%	
**Major location**					<0.001					0.876					0.740
Cardia	382	35.0%	32	23.7%		74	20.2%	25	18.1%		56	25.8%	7	21.9%	
Body	215	19.7%	35	25.9%		76	20.8%	27	19.6%		40	18.4%	5	15.6%	
Antrum	451	41.4%	51	37.8%		197	53.8%	77	55.8%		117	53.9%	20	62.5%	
Whole	42	3.9%	17	12.6%		19	5.2%	9	6.5%		4	1.8%	0	0	
**Biopsy differentiation**					0.019					<0.001					0.053
Well / Moderate	202	18.5%	14	10.4%		140	38.3%	29	21.0%		86	39.6%	7	21.9%	
Poor	888	81.5%	121	89.6%		226	61.7%	109	79.0%		131	60.4%	25	78.1%	
**Lauren type**					<0.001					<0.001					0.006
Intestinal / mixed	716	65.7%	68	50.4%		280	76.5%	71	51.4%		160	73.7%	16	50.0%	
Diffuse	374	34.3%	67	49.6%		86	23.5%	67	48.6%		57	26.3%	16	50.0%	
**CEA**					0.026					0.062					0.487
Normal	887	81.4%	99	73.3%		45	12.3%	9	6.5%		22	10.1%	2	6.3%	
Elevated	203	18.6%	36	26.7%		321	87.7%	129	93.5%		195	89.9%	30	93.8%	
**CA199**					0.014					0.153					0.450
Normal	888	81.5%	98	72.6%		52	14.2%	13	9.4%		31	14.3%	3	9.4%	
Elevated	202	18.5%	37	27.4%		314	85.8%	125	90.6%		186	85.7%	29	90.6%	
**Clinical T stage**					0.078					0.406					0.498
T2/T3	304	27.9%	28	20.7%		112	30.6%	37	26.8%		88	40.6%	15	46.9%	
T4	786	72.1%	107	79.3%		254	69.4%	101	73.2%		129	59.4%	17	53.1%	
**Clinical N stage**					0.088					0.747					0.194
N0	396	36.3%	39	28.9%		157	42.9%	57	41.3%		76	35.0%	15	46.9%	
N+	694	63.7%	96	71.1%		209	57.1%	81	58.7%		141	65.0%	17	53.1%	

© 2021 Jiang Y et al. *JAMA Network Open.*
